# Comorbidity Patterns of Mood Disorders in Adult Inpatients: Applying Association Rule Mining

**DOI:** 10.3390/healthcare9091155

**Published:** 2021-09-03

**Authors:** Sunkyung Cha, Sung-Soo Kim

**Affiliations:** 1 Department of Nursing Science, Sunmoon University, Asan 31460, Korea; skc0701@hanmail.net; 2 Department of Health Administration & Healthcare, Cheongju University, Cheongju 28503, Korea

**Keywords:** mood disorder, comorbidity, ARM (association rule mining), Korean national hospital discharge in-depth injury survey (KNHDS)

## Abstract

This study explored physical and psychiatric comorbidities of mood disorders using association rule mining. There were 7709 subjects who were patients (≥19 years old) diagnosed with mood disorders and included in the data collected by the Korean National Hospital Discharge In-depth Injury Survey (KNHDS) between 2006 and 2018. Physical comorbidities (46.17%) were higher than that of psychiatric comorbidities (27.28%). The frequent comorbidities of mood disorders (F30–F39) were hypertensive diseases (I10–I15), neurotic, stress-related and somatoform disorders (F40–F48), diabetes mellitus (E10–E14), and diseases of esophagus, stomach, and duodenum (K20–K31). The bidirectional association path of mood disorders (F30–F39) with hypertensive diseases (I10–I15) and diabetes mellitus (E10–E14) were the strongest. Depressive episodes (F32) and recurrent depressive disorders (F33) revealed strong bidirectional association paths with other degenerative diseases of the nervous system (G30-G32) and organic, including symptomatic and mental disorders (F00–F09). Bipolar affective disorders (F31) revealed strong bidirectional association paths with diabetes mellitus (E10–E14) and hypertensive diseases (I10–I15). It was found that different physical and psychiatric disorders are comorbid according to the sub-classification of mood disorders. Understanding the comorbidity patterns of major comorbidities for each mood disorder can assist mental health providers in treating and managing patients with mood disorders.

## 1. Introduction

Mood disorders, including depressive disorders and bipolar disorders, are common mental disorders in mental health practice. Depressive disorders are one of the major causes of the years lived with disability. Moreover, depression and anxiety have incurred a loss of USD 1 trillion to the world economy annually [1,2,3]. Mood disorders are a mental disorder type that burdens individuals and society highly.

The research analyzed the health insurance claims of mood disorders (F30–F39) from 2016 to 2020 using the medical data of the National Health Service of South Korea and reported that the number of patients with mood disorders increased by 6.9% annually on average [4]. Furthermore, the majority of mental disorder patients are hospitalized due to mood disorders [5]. In South Korea, the total health insurance of claims due to mood disorders increased by 57.2% (KRW 245.9 billion) over five years from KRW 429.9 billion in 2016 to KRW 675.7 billion in 2020, with an average annual increase rate of 12.0% [4].

The most common mood disorder is major depressive disorder, and the lifetime prevalence of the major depressive disorder is between 4.4% and 30%. National Health Insurance Services analyzed a large sample and reported that the prevalence of depression in South Korea increased from 2.8% in 2002 to 5.3% in 2013 [6]. Since the outbreak of the COVID-19 pandemic, the incidence of depression has increased at least two-fold worldwide. Particularly, as of 2020, the prevalence of depression in South Korea was 36.8%, the highest among OECD member countries [7].

The lifetime prevalence of bipolar disorders, the second most common disorder type after depressive disorders, has been reported as between 0.5% and 2.5%. Bipolar disorders are relatively common mental disorders that may require lifelong treatment. Previous studies reported that the number of patients treated due to bipolar disorders increased by 21.0% over five years (2013–2017). The medical service fee for patients with bipolar disorders also increased by KRW 17 billion (19.5%), from KRW 87.2 billion in 2013 to KRW 104.2 billion in 2017, and the cost of treatment per hospitalized patient increased by 4.6% over the past five years on average [8].

The prevalence and medical expenses of mental disorders, including mood disorders, have been increasing due to an accelerated aging trend, the complexity of the social environment, and the emergence of various infectious diseases. Moreover, many studies [4,5,6,7,8] have reported aggravated symptom burden, functional impairment, decreased quality of life, and increased cost, especially when a mental disorder and a mental or physical problem are accompanied. The registration of comorbid diseases is also related to the increase in survival rate. It is an important research topic that needs to be studied continuously according to national and international disease classification standards and subjects. It was found that mood disorders, including depressive disorders, were most often accompanied by other mental or chronic diseases [9].

However, only a few studies on the comorbidities of mental disorders explored the patterns of physical and psychological comorbidities of hospitalized patients while focusing on the sub-diagnosis of mood disorders that greatly influence individuals and society. In terms of analysis methods, there are only a few studies that utilized ARM (association rule mining), which were used in studies on comorbid diseases in the health care field based on clinical datasets [5,10,11].

The objective of this study was to explore the patterns of physical and mental comorbidities in hospitalized mood disorder patients by applying the association rule analysis. We hope that the results of this study can lay out a foundation for treating and managing patients with a mood disorder in the clinical setting by identifying the association pattern between individual mood disorders and comorbidities.

## 2. Materials and Methods

### 2.1. Data Source

This study used the data of the Korean National Hospital Discharge In-depth Injury Survey (KNHDS). The KNHDS is being carried out to provide basic data necessary for policymaking by producing continuous national health statistics on major chronic diseases and injuries using the medical records of discharged patients. The KNHDS is a nationally approved statistic conducted annually by the Korea Centers for Disease Control and Prevention. Currently, 14 years of data from 2005 to 2018 have been refined and released to the public [12]. The survey population of the KNHDS was patients who were hospitalized and discharged from general hospitals with more than 100 beds, excluding single department hospitals, nursing hospitals, senior hospitals, and veteran’s hospitals, based on the discharge date of the previous year. The KNHDS used the stratified two-stage cluster sampling method. The first stage was individual hospitals belonging to the survey population, and the extraction unit in the second stage was discharged patients. The number of beds was classified into four tiers, and it was used as a stratification variable. Survey items included demographic information (e.g., gender and age) and clinical information (e.g., principal diagnosis, additional diagnosis, surgery, and treatment). Principal diagnoses were classified by using the ICD-10-based KCD-7 code, while surgeries and treatments were classified according to the ICD-9-CM code. The Korea Centers for Disease Control and Prevention provides data to researchers after excluding variables that may reveal the identity of individuals as prescribed by the privacy law. 

### 2.2. Study Population

The subjects of this study were discharged patients whose principal diagnosis was mood disorders (F30–F39) in the KNHDS from 2006 to 2018. This study excluded 2005 data, which recorded up to ten additional diagnoses. Since the 2006 survey, the KNHDS has recorded up to 20 additional diagnoses. This study analyzed 7709 discharged patients.

### 2.3. Principal Diagnosis & Comorbidities

The main variables of this study were the main diagnosis and comorbidities. The principal diagnosis is the final diagnosis confirmed after conducting examinations, and it is the main reason for admission [13,14]. Only one disease is registered, and most medical resources are applied due to this disease during hospitalization [15]. This study used the additional diagnosis of the KNHDS data for analyzing comorbidities. The comorbidities refer to various diseases diagnosed during hospitalization in addition to the principal disease. The number of comorbidities depends on the patient: some patients did not have any, and some patients had more than one [16,17]. Principal diagnosis and comorbidities were applied as the middle classification criteria of Korean standard classification of diseases (KCD, 7th) based on the International Classification of Diseases, Tenth Revision (ICD-10). The data was divided into adult psychiatric comorbidity (F00–F69) and physical comorbidity (A00–Z99) (excluding the psychiatric comorbidities) to confirm the detailed comorbidity prevalence.

### 2.4. Statistical Analysis

The raw data of KNHDS used in this study is a big dataset containing more than 2.7 million cases. This study built a database using MySQL in order to efficiently manage the data. After extracting data using Structured Query Language (SQL) according to the subject selection criteria, R (version 4.1.0, R Foundation for Statistical Computing, Vienna, Austria) was used for analyzing the data. The χ^2^-test was conducted to analyze the prevalence and distribution of comorbidities by demographic characteristics and principal diagnosis. Moreover, ARM was used to explore the relationship patterns between comorbidities. arulesViz was used for the visualization of association rules, and the algorithm used for ARM was Apriori. Apriori is a representative algorithm designed to focus on the occurrence of events, and it makes it easy to analyze the comorbidities of the target dataset [18,19]. The application of it has been expanded to medical fields, such as clinical decision-making to diagnose diseases in recent years [20,21,22]. It is also known as an unsupervised machine learning method. Support, confidence, and lift were used as the evaluation indicator of ARM analysis results. Although lift is mainly used for presenting highly correlated association rules, this study additionally used the interest support (IS) scale.

In ARM, support is the ratio of the number of transactions that include both X and Y out of the total number of transactions. Reliability is the proportion of transactions involving X (conditional probability) that also includes Y. The improvement is the ratio of the probability of Y when X occurs to the probability of Y when X does not occur. X and Y were replaced with comorbid diseases A and B and applied to this study. In this study, the support indicated the ratio of two or more comorbidities diagnosed together during hospitalization. The confidence refers to the ratio of patients diagnosed with comorbidity B among those diagnosed with comorbidity A. Lift is a scale indicating the correlation of the association rules: the ratio of the probability of diagnosing comorbidity A and comorbidity B at the same time compared to the probability of diagnosing comorbidity A or comorbidity B independently [23]. The IS scale is used to find more meaningful association rules by filtering out association rules that have either high support or high lift [24]. The study extracted initial association rules while considering the data characteristics (many comorbidity classification categories and large sample size) that satisfied support >1% and confidence >10%. Additionally, this study applied the condition of lift >1 to select only association rules showing high correlation. Afterward, the rules are presented in order of IS scale.

(1) $$\mathrm{Support}\left(A\to B\right)=\frac{Number\;of\;patients\;with\;A\;and\;B}{Total\;number\;of\;patients}$$

(2) $$\mathrm{Confidence}\left(A\to B\right)=\frac{Number\;of\;patients\;with\;A\;and\;B}{Number\;of\;patients\;with\;A}$$

(3) $$\mathrm{Lift}\left(A\to B\right)=\frac{Support\left(A\;\to \;B\right)}{P\left(comorbidity\;A\right)\times P\left(comorbidity\;B\right)}$$

(4) $$\mathrm{IS}\left(A\to B\right)=\sqrt{\mathrm{Support}\left(A\to B\right)\times \mathrm{Lift}\left(A\to B\right)}$$

## 3. Results

### 3.1. Prevalence of Comorbidities in Study Population

Table 1 shows the analysis results of the prevalence of psychiatric comorbidity and physical comorbidity by demographic characteristics. There were 7709 patients whose principal diagnosis was a mood disorder, and 60.6% (4630 patients) had comorbidities. The proportion of psychiatric comorbidity was 27.28%, and that of physical comorbidity was 46.17%, higher than psychiatric comorbidity. The proportion of psychiatric comorbidity was 29.97% for males and 26.09% for females, and males had psychiatric comorbidity significantly (*p* < 0.001) more than females. On the other hand, the proportion of physical comorbidity was similar between females (46.46%) and males (45.51%) (*p* = 0.444). The rate of physical comorbidity increased as the age of subjects increased (*p* < 0.001). However, the rate of psychiatric comorbidity was in the order of 75 years or older (29.26%), 19–44 years old (28.48%), and 45–64 years old (26.26%) (*p* = 0.039). In insurance type, the prevalence rate of psychiatric comorbidity was in the order of others (50.00%), Medicaid II (34.31%), Medicaid I (31.35%), and National health (26.48%) (*p* < 0.001). The prevalence rate of physical comorbidity was in the order of Medicaid I (51.43%), others (48.44%), and National health (45.82%) (*p* = 0.081). Outpatient (28.50%) was the most common admission route for psychiatric comorbidity, while emergency (47.58%) was the most common type for physical comorbidity.

The prevalence of comorbidity by principal mood disorder diagnosis was analyzed (Table 2). The prevalence of psychiatric comorbidity was in the order of other mood disorders (42.86%), persistent mood disorders (41.88%), unspecified mood disorders (37.04%), and depressive episodes (31.12%) (*p* < 0.001). The prevalence of physical comorbidity was in the order of other mood disorders (57.14%), recurrent depressive disorders (51.63%), depressive episodes (50.67), and manic episodes (46.67%) (*p* < 0.001).

### 3.2. Frequency of Comorbidities

**Figure 1:**
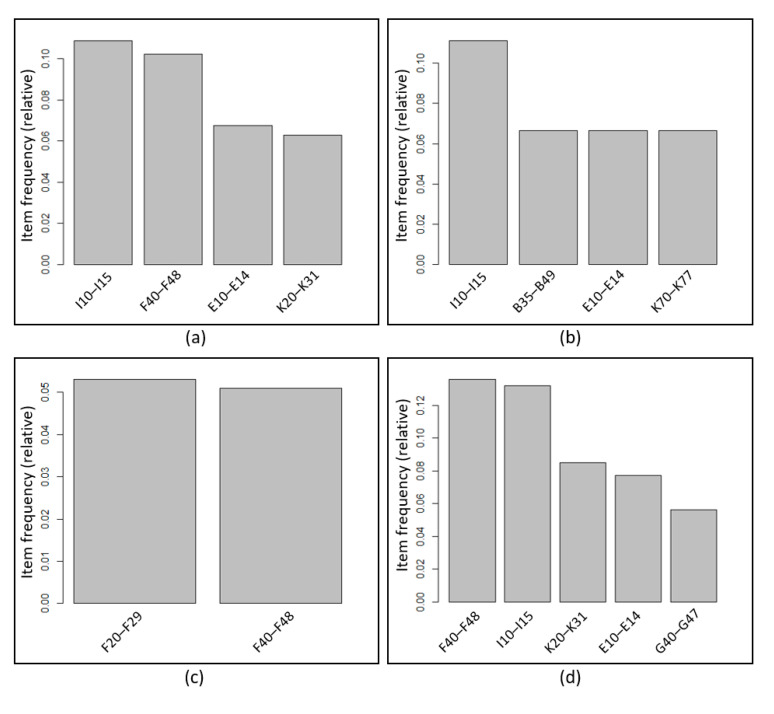

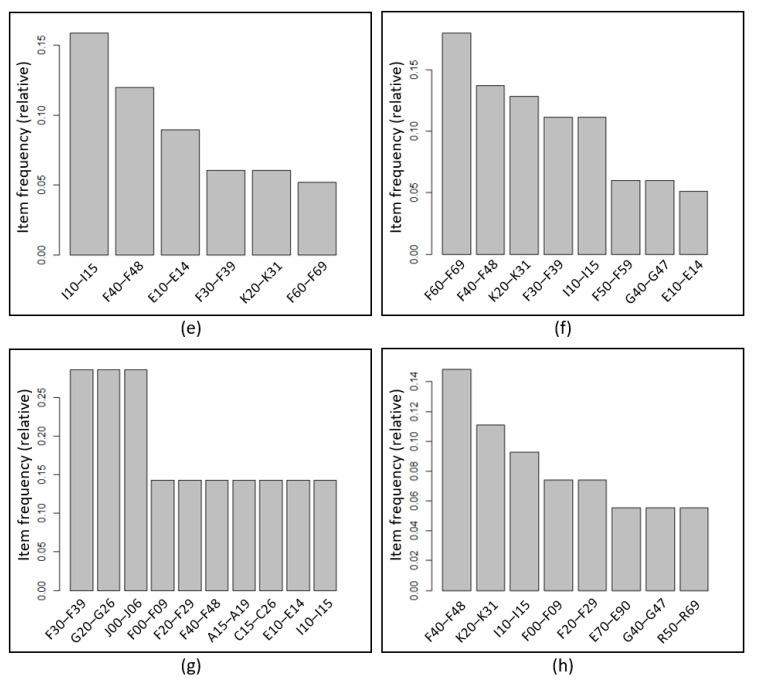
Frequent comorbidities by sub-diagnosis of mood disorders. (**a**) Mood disorders (F30–F39), (**b**) manic episode (F30), (**c**) bipolar affective disorders (F31), (**d**) depressive episode (F32), (**e**) recurrent depressive disorders (F33), (**f**) persistent mood disorders (F34), (**g**) other mood disorders (F38), (**h**) unspecified mood disorders (F39). ICD–10 code: A15–A19, tuberculosis; B35–B49, mycoses; C15–C26, malignant tumor of digestive organs; E10–E14, diabetes mellitus; E70–E90, metabolic disorders; F00–F09, organic, including symptomatic, mental disorders; F20–F29, schizophrenia, schizotypal, and delusional disorders; F30–F39, mood disorders; F40–F48, neurotic, stress-related, and somatoform disorders; F50–F59, behavioral syndromes associated with physiological disturbances and physical factors; F60–F69, disorders of adult personality and behavior; G20–G26, extrapyramidal and movement disorders; G40–G47, episodic and paroxysmal disorders; I10–I15, hypertensive diseases; J00–J06, acute upper respiratory infections; K20–K31, diseases of esophagus, stomach, and duodenum; K70–K77, diseases of liver; R50–R69, general symptoms and signs.

The relative frequencies of comorbidities with support > 5% were analyzed for each subclass of the principal diagnosis (Figure 1). The most frequent comorbidities of patients with a mood disorder (F30–F39) as a principal diagnosis were hypertensive diseases (I10–I15), neurotic, stress-related and somatoform disorders (F40–F48), diabetes mellitus (E10–E14), and diseases of the esophagus, stomach, and duodenum (K20–K31). The most frequent comorbidities of a manic episode (F30) were hypertensive diseases (I10–I15), mycoses (B35–B49), diabetes mellitus (E10–E14), and diseases of the liver (K70–K77). The most frequent comorbidities of bipolar affective disorders (F31) were schizophrenia, schizotypal and delusional disorders (F20–F29), neurotic, and stress-related and somatoform disorders (F40–F48), all of which were psychiatric comorbidities. The most frequent comorbidities of a depressive episode (F32) were in the order of neurotic, stress-related and somatoform disorders (F40–F48), hypertensive diseases (I10–I15), diseases of the esophagus, stomach, and duodenum (K20–K31), diabetes mellitus (E10–E14), and episodic and paroxysmal disorders (G40–G47). Recurrent depressive disorders (F33) included hypertensive diseases (I10–I15), neurotic, stress-related and somatoform disorders (F40–F48), diabetes mellitus (E10–E14), mood disorders (F30–F39), and diseases of the esophagus, stomach, and duodenum (K20–K31). The frequent comorbidities of persistent mood disorders (F34) are in the order of disorders of adult personality and behavior (F60–F69), neurotic, stress-related and somatoform disorders (F40–F48), diseases of the esophagus, stomach, and duodenum (K20–K31), mood [affective] disorders (F30–F39), and hypertensive diseases (I10–I15). The frequent comorbidities of other mood disorders (F38) were mood [affective] disorders (F30–F39), extrapyramidal and movement disorders (G20–G26), acute upper respiratory infections (J00–J06), and organic, including symptomatic, mental disorders (F00–F09). The frequent comorbidities of unspecified mood disorders (F39) were neurotic, stress-related and somatoform disorders (F40–F48), diseases of the esophagus, stomach, and duodenum (K20–K31), hypertensive diseases (I10–I15), and organic, including symptomatic, mental disorders (F00–F09).

It was found that hypertensive diseases (I10–I15) were commonly comorbid diseases by patients, except for those diagnosed with bipolar affective disorder (F31) as a principal diagnosis. Diabetes mellitus (E10–E14) was frequently a comorbid disease in patients, except for those diagnosed with a bipolar affective disorder (F31) and an unspecified mood disorder (F39) as a principal diagnosis. Neurotic, stress-related and somatoform disorders (F40–F48) among psychiatric comorbidities showed the characteristic of being comorbid with the exception of patients whose principal diagnosis was a manic episode (F30).

### 3.3. Association Rules among Comorbidities

**Figure 2:**
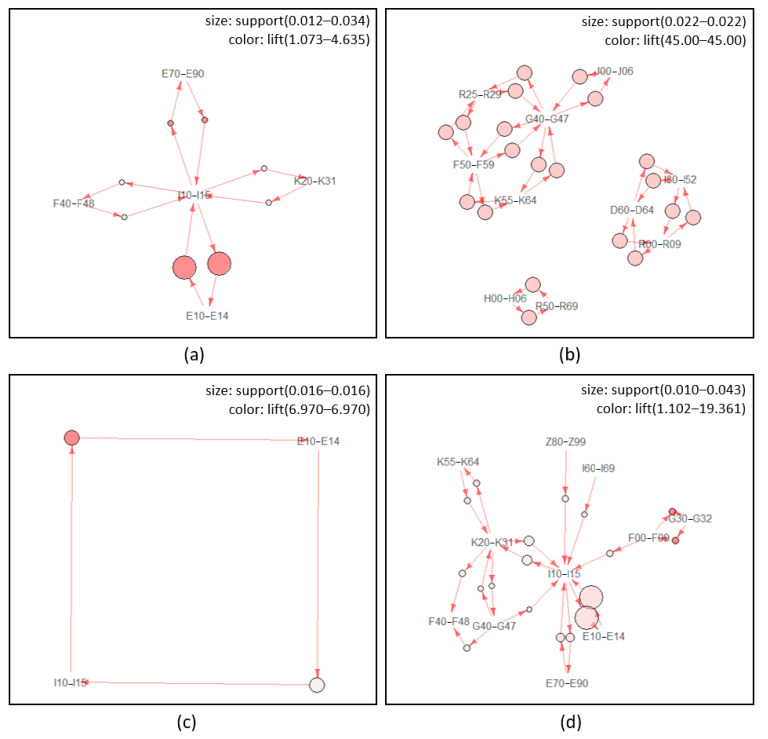

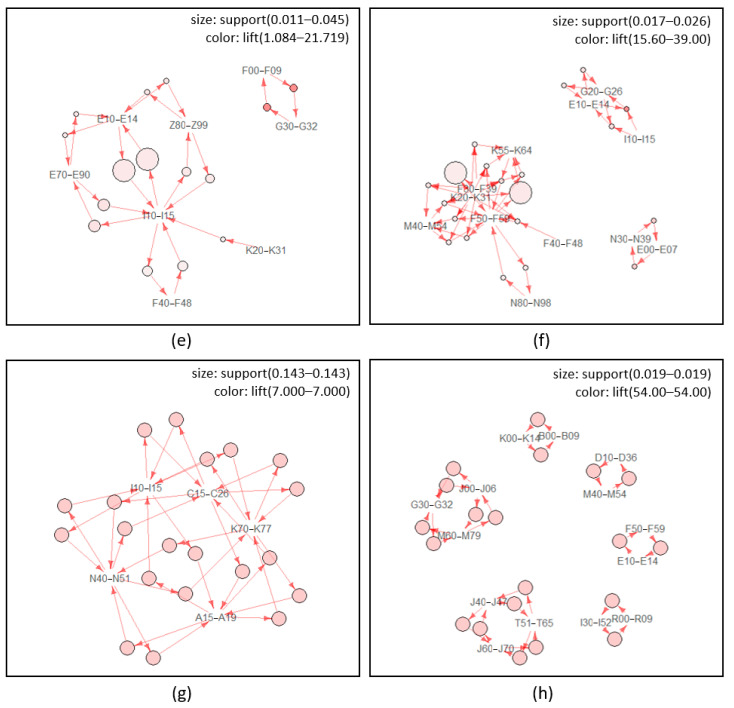
Network graph for comorbid diseases of mood disorders inpatients. (**a**) Mood disorders (F30–F39), (**b**) manic episode (F30), (**c**) bipolar affective disorders (F31), (**d**) depressive episode (F32), (**e**) recurrent depressive disorders (F33), (**f**) persistent mood disorders (F34), (**g**) other mood disorders (F38), (**h**) unspecified mood disorders (F39). ICD–10 code: A15-A19, tuberculosis; B00–B09, viral infections characterized by skin and mucous membrane lesions; C15–C26, digestive organs; D10–D36, benign neoplasms; D60–D64, aplastic and other anemias; E00–E07, disorders of thyroid gland; E10–E14, diabetes mellitus; E70–E90, metabolic disorders; F00–F09, organic, including symptomatic, mental disorders; F30–F39, mood disorders; F40–F48, neurotic, stress-related, and somatoform disorders; F50–F59, behavioral syndromes associated with physiological disturbances and physical factors; G20–G26, extrapyramidal and movement disorders; G30–G32, other degenerative diseases of the nervous system; G40–G47, episodic and paroxysmal disorders; H00–H06, disorders of eyelid, lacrimal system, and orbit; I10–I15, hypertensive diseases; I30–I52, other forms of heart disease; I60–I69, cerebrovascular diseases; J00–J06, acute upper respiratory infections; J40–J47, chronic lower respiratory diseases; J60–J70, lung diseases due to external agents; K00–K14, diseases of oral cavity, salivary glands, and jaws; K20–K31, diseases of esophagus, stomach, and duodenum; K55–K64, other diseases of intestines; K70–K77, diseases of liver; M40–M54, dorsopathies; M60–M79, soft tissue disorders; N30–N39, other diseases of the urinary system; N40–N51, diseases of male genital organs; N80–N98, noninflammatory disorders of female genital tract; R00–R09, symptoms and signs involving the circulatory and respiratory systems; R25–R29, symptoms and signs involving the nervous and musculoskeletal systems; R50–R69, general symptoms and signs; T51–T65, toxic effects of substances chiefly nonmedicinal as to source; Z80–Z99, persons with potential health hazards related to family and personal history and certain conditions influencing health status.

Figure 2 shows the schematic diagram of association paths by using network graphs for each subgroup of mood disorders. This study visualized up to 20 association paths because it is difficult to recognize them visually when too many paths are displayed. A larger circle indicates greater support, and darker color means the strength of lift. Mood disorders (F30–F39) formed association paths with diabetes mellitus (E10–E14) and other comorbidities centered on hypertensive diseases (I10–I15). Manic episodes (F30) formed a large association path group centered on behavioral syndromes associated with physiological disturbances and physical factors (F50–F59) and episodic and paroxysmal disorders (G40–G47). Bipolar affective disorders (F31) showed only the bidirectional paths with hypertensive diseases (I10–I15) and diabetes mellitus (E10–E14). Hypertensive diseases (I10–I15) form the central axis in the depressive episode (F32). It was found that recurrent depressive disorders (F33) had associated paths with comorbidities around diabetes mellitus (E10–E14) and hypertensive diseases (I10–I15). No special characteristic was found in the rest of the mood disorders.

Table 3 shows the results of exploring the paths of major comorbidities (≤ 5) for each principal diagnosis using support, confidence, lift, and IS scales. It was found that the bidirectional association path of mood disorders (F30–F39) with diabetes mellitus (E10–E14) and hypertensive diseases (I10–I15) were the strongest with an IS scale of 0.397. Afterward, the analysis results revealed that the bidirectional association path between metabolic disorders (E70–E90) and hypertensive diseases (I10–I15) were 0.234 (IS scale). Manic episodes (F30) showed strong bidirectional association paths (IS scale = 1.000) with general symptoms and signs (R50–R69), disorders of the eyelid, lacrimal system, and orbit (H00–H06), aplastic and other anemias (D60–D64), and other forms of heart disease (I30–I52). Bipolar affective disorders (F31) revealed strong bidirectional association paths (IS scale = 0.331) with diabetes mellitus (E10–E14) and hypertensive diseases (I10–I15). The strength of depressive episode’s (F32) bidirectional association path were in the order of that between other degenerative diseases of the nervous system (G30–G32) and organic, including symptomatic and mental disorders (F00–F09) (IS scale = 0.496), and that between diabetes mellitus (E10–E14) and hypertensive diseases (I10–I15) (IS scale = 0.425). The strength of recurrent depressive disorders’ (F33) bidirectional association path were in the order of that between other degenerative diseases of the nervous system (G30–G32) and organic, including symptomatic, mental disorders (F00–F09) (IS scale = 0.584), and that between metabolic disorders (E70–E90) and hypertensive diseases (I10–I15) (IS scale = 0.324). Persistent mood disorders (F34) showed the strongest association path from diabetes mellitus (E10–E14) and hypertensive diseases (I10–I15) to extrapyramidal and movement disorders (G20–G26) (IS scale = 0.816), followed by the bidirectional association path between disorders of the thyroid gland (E00–E07) and other diseases of the urinary system (N30–N39) (IS scale = 0.667), and extrapyramidal and movement disorders (G20–G26) and diabetes mellitus (E10–E14) (IS scale = 0.577). In other mood disorders (F38), the bidirectional association path (IS scale = 1.000) between tuberculosis (A15–A19) and digestive organs (C15–C26), tuberculosis (A15–A19) and that (IS scale = 1.000) between tuberculosis (A15–A19) and hypertensive diseases (I10–I15) were equal. In unspecified mood disorders (F39), the bidirectional association path (IS scale = 1.000) between viral infections characterized by skin and mucous membrane lesions (B00–B09) and diseases of oral cavity, salivary glands, and jaws (K00–K14) and that (IS scale = 1.000) between benign neoplasms (D10–D36) and dorsopathies (M40–M54) were equal.

## 4. Discussion

Mood disorders, including depressive disorders, have received high attention worldwide, and the prevalence and medical expenses of mood disorders are increasing in South Korea. In addition, the comorbidity of a mental disease is an important factor that affects the duration of hospitalization, survival rate, and medical service utilization [9]. Therefore, this study aimed to examine major physical and psychiatric comorbidities according to each sub-diagnosis and explore the association rules for comorbidities using patients hospitalized due to mood disorders using the KNHDS data.

Patients hospitalized due to mood disorders had physical comorbidities more often than psychiatric comorbidities. Although the prevalence of physical comorbidities was not different between genders, it increased with older age. The prevalence of psychiatric comorbidities was significantly higher in male subjects than in female subjects, and it was in the order of 75 or older, 19–44 years old, and 45–64 years old. Further, it was lower among patients admitted to hospitals with more than 1000 beds. This is related to the fact that hospitals with more than 1000 beds have high treatment costs among acute-stage mental health treatment institutions and patients who are hospitalized in the early stages of the disease process discharged relatively quickly after treatment of the main diagnosis.

The comorbidity rate of all mood disorders was higher in male subjects than in female subjects, and the rate was proportional to age. It is believed that the results of this study are related to [9], who reported that the prevalence of complex mental disorders (cases of suffering from two or more mental disorders or a chronic disease and at least one mental disorder) was higher with female subjects than male subjects, but female subjects used medical services less than male subjects per year and their co-payment was lower than male subjects’ co-payment and argued that it was related to the socioeconomic status of women and gender roles. It is difficult to make a direct comparison because there is no study on the comorbidity prevalence of hospitalized mood disorder patients. Therefore, future studies need to measure expanded study subjects repetitively.

The principal sub-diagnosis of hospitalized mood disorder patients was more frequent in the order of depressive episodes (F32), bipolar affective disorders (F31), and recurrent depressive disorders (F33), which was somewhat different from the number of treated mood disorder patients, including outpatient departments, in South Korea in 2020, the order was: depressive episode (F32), bipolar affective disorders (F31), persistent mood disorders (F34), and recurrent depressive disorders (F33) [25]. It could be because, although the diagnostic criteria for persistent mood disorders were longer than a depressive episode (F32) or recurrent depressive disorders (F33), fewer patients with persistent mood disorders was hospitalized due to mild symptoms. It seems that the dataset including outpatients and the hospitalized patient dataset have different characteristics when evaluating comorbidities, and further studies are needed.

The common frequently observed physical comorbidities of sub-diagnosis of mood disorders were hypertensive diseases (I10–I15) and diabetes mellitus (E10–E14), while the commonly observed psychiatric comorbidity of them was neurotic, stress-related, and somatoform disorders (F40–F48), including severe anxiety disorder and somatic disorders. These results agreed with a study on comorbidities of all psychiatric disorders [5], and they were related to the results of previous studies showing that the comorbidity rate of anxiety disorders was high when patients suffered from a mood disorder or bipolar disorder [26,27]. In addition, although the specific and frequent comorbidities of each sub-diagnosis of mood disorder need to be studied further, they seem meaningful. For psychiatric comorbidity examples, schizophrenia, schizotypal, and delusional disorders (F20–F29) accompanied by bipolar affective disorders (F31) and disorders of adult personality and behavior (F60–F69) accompanied by persistent mood disorders (F34). It is hard to study psychiatric comorbidities in mood disorders because of the coexistence of a bipolar disorder, and another psychiatric disorder complicates psychiatric diagnosis and treatment, and the overlap of symptoms makes it hard to define and recognition of true comorbidities when diagnosing a disease with the Diagnostic and Statistical Manual of Mental Disorders [28]. Therefore, it needs to be studied continuously. In the case of physical comorbidities, examples are the comorbidity of mycoses (B35–B49) related to lack of self-care in manic episodes (F30) or else other comorbidities related to clinical characteristics of an individual mental disorder.

In terms of the pattern of association rule by mood disorder sub-diagnosis, mood disorders (F30–F39) were closely correlated with the bidirectional paths of diabetes mellitus (E10–E14) and hypertensive diseases (I10–I15) and those of metabolic disorders (E70–E90) and hypertensive diseases (I10–I15). The results of this study were somewhat different from the results of a previous study [5], showing a strong correlation with the bidirectional paths of family and personal history and certain conditions influencing health status (Z80–Z99) and hypertensive diseases (I10–I15) and those of hypertensive diseases (I10–I15) and diseases of the esophagus, stomach, and duodenum (K20–K31). Although the five major paths with high IS scale values were rather different, the schematic diagram depicting 20 associations showed that they were similar except for the paths of hypertensive diseases (I10–I15) in family and personal history and certain conditions influencing health status (Z80–Z99). This could be partly because they include the same subjects.

Manic episodes (F30) showed a strong correlation with the association between general symptoms and signs (R50–R69) and disorders of eyelid, lacrimal system, and orbit (H00–H06), the association between aplastic and other anemias (D60–D64) and other forms of heart disease (I30–I52), and the path of symptoms and signs involving the circulatory and respiratory systems (R00–R09) in aplastic and other anemias (D60–D64). It is believed that it is related to the fact that the anticonvulsants and antipsychotic drugs used for treating manic episodes can cause aplastic anemia [29].

It was found that only diabetes mellitus (E10–E14) and hypertensive diseases (I10–I15), physical comorbidities, were significant comorbidities of bipolar affective disorders (F31). The result is related to the findings of [30], who showed that adults with bipolar I disorder experienced an increased risk of CVD and HTN at least 10 years earlier than adults with non-bipolar I disorder, and a strategy would be needed to prevent the cardiovascular burden of patients with bipolar I disorder. A literature review on comorbidities of bipolar disorders has reported that psychiatric complications were often associated with earlier onset of bipolar symptoms, a more severe course, poorer treatment adherence, and worse outcomes associated with suicide and other complications and that medical comorbidities could be exacerbated to bipolar symptoms or be induced by drug therapy for bipolar symptoms [28]. Although it is often unclear whether a medical condition is truly accompanied, a result of treatment, or a combination of both, it is clear that it is necessary to observe the comorbidity of diabetes mellitus (E10–E14) and hypertensive diseases (I10–I15) and reflect the observation to treatment and management. This is because the accompaniment of these physical diseases can influence the functional level of cognitive ability and others in the future [31].

The results of the depressive episode (F32) revealed that it is needed to carefully examine the comorbidity of other degenerative diseases of the nervous system (G30–G32) and organic, including symptomatic, mental disorders (F00–F09), such as dementia. A study suggested that patients with epilepsy, Parkinson’s disease, and ischemic stroke frequently showed depression and suicide, and it is necessary to pay attention to accompanying psychiatric disorders when there is a neurological disease [32]. Another study argued that depression was a risk factor for Alzheimer’s disease and dementia symptoms [33]. The comorbidity of diabetes mellitus (E10–E14) and hypertensive diseases (I10–I15) also needs to be carefully observed. Several studies have provided empirical evidence for the comorbidities of depression, hypertension, and diabetes: depressive mood and cardiovascular diseases were frequently found in approximately 20 to 45% of people with heart disease and depression [34]; depression increased diabetes by 60%, and diabetes increased depression by 15% [35]; diabetic patients had a higher risk of coronary heart disease [36]; and major depressive disorders increased the incidence of cardiovascular disease in diabetic patients [37].

Recurrent depressive disorders (F33) need to manage the accompaniment of other degenerative diseases of the nervous system (G30–G32) and organic, including symptomatic, mental disorders (F00–F09), and the accompaniment of metabolic disorders (E70–E90) and hypertensive diseases (I10–I15). In general, diabetes, thyroid diseases, liver diseases, and kidney diseases are considered in the selection of antidepressants according to the physical disease accompanying depression [38]. It is necessary to secure evidence regarding depression-related disorders that have been studied much less than major depressive disorders through replication studies.

Persistent mood disorders (F34) showed that diabetes mellitus (E10–E14) and hypertensive diseases (I10–I15), unlike other mood disorders, had paths related to extrapyramidal and movement disorders (G20–G26), disorders of the thyroid gland (E00–E07), and other diseases of the urinary system (N30–N39). Other mood disorders (F38) had paths between tuberculosis (A15–A19) and digestive organs (C15–C26), in addition to hypertensive diseases (I10–I15). Unspecified mood disorders (F39) showed the accompaniment of viral infections characterized by skin and mucous membrane lesions (B00–B09) and diseases of the oral cavity, salivary glands, and jaws (K00–K14) and that of benign neoplasms (D10–D36) and dorsopathies (M40–M54). Therefore, this study presented the analysis results even when the size of the antecedent was small because this study did not evaluate causality. Although it is necessary to interpret the results carefully and the results are sorted according to the IS scale, high support values could be important. The principal sub-diagnoses of mood disorders differed between frequently observed physical and psychiatric comorbidities, and the association rules between comorbidities also had unique characteristics.

## 5. Conclusions

The patterns of comorbidities in patients with mood disorders were evaluated using ARM based on clinical data, and it was found that different physical and psychiatric disorders comorbid each sub-diagnosis of mood disorders. Consequently, clinicians need to be aware of major comorbidities for each sub-diagnosis when treating and managing patients with mood disorders. Moreover, it is necessary to explain and educate this to patients and their families. It is expected that the results of this study will lay a foundation for the treatment and management of comorbidities in patients with mood disorders. It will be necessary to conduct replication studies with the expanding of subjects of sub-diagnosis of mood and continue to evaluate disorders and other psychiatric disorders, such as schizophrenia and anxiety disorders. There is also a need for pursuing political efforts in the mental health practice, such as establishing a system for registering and managing major comorbidities of mental disorders, preparing a cooperative system for managing comorbidities, and using them in clinical treatment and management. The importance of this study was to evaluate the physical and psychiatric comorbidities of hospitalized mood disorder patients using the systematically collected national data, explore the patterns of association rules, and provide clinical implications. However, this study only evaluated patients hospitalized in general hospitals with more than 100 beds, and it could not determine whether the comorbidities were diagnosed during hospitalization or if they were pre-existing conditions, which were the limitations of this study. Moreover, there is a limitation in generalizing the results because this study is based on large-scale clinical data collected in South Korea.

## Figures and Tables

**Table 1:** General characteristics of study population and prevalence of comorbidities.

Variables	Psychiatric Comorbidity ^a^		Physical Comorbidity ^b^		Total ^c^	
	*N*(%)	χ^2^ (*p*)	*N*(%)	χ^2^ (*p*)	*N*(%)	χ^2^ (*p*)
Sex		12.466		0.587		5.476
Male	708(29.97)	(<0.001)	1075(45.51)	(0.444)	1465(62.02)	(0.019)
Female	1395(26.09)		2484(46.46)		3165(59.19)	
Age group		8.385		482.304		222.946
19–44	983(28.48)	(0.039)	1170(33.89)	(<0.001)	1791(51.88)	(<0.001)
45–64	670(26.26)		1270(49.78)		1593(62.45)	
65–74	275(24.82)		719(64.89)		800(72.2)	
≥ 75	175(29.26)		400(66.89)		446(74.58)	
Insurance type		42.203		6.720		22.724
National health	1851(26.48)	(<0.001)	3203(45.82)	(0.081)	4156(59.45)	(<0.001)
Medicaid I	153(31.35)		251(51.43)		316(64.75)	
Medicaid II	35(34.31)		43(42.16)		59(57.84)	
Others	64(50.00)		62(48.44)		99(77.34)	
Admission route		N/A		N/A		1.249
Emergency	525(24.18)		1033(47.58)		1289(59.37)	(0.535)
Outpatient	1578(28.50)		2525(45.60)		3340(60.32)	
Others	-		1(100)		1(100)	
Treatment outcome		2.398		N/A		21.233
Improved	1922(27.19)	(0.494)	3312(46.85)		4273(60.45)	(<0.001)
Not improved	174(28.20)		229(37.12)		335(54.29)	
Death	1(12.50)		8(100.00)		8(100)	
Others	6(40.00)		10(66.67)		14(93.33)	
Number of hospital beds		33.952		21.480		23.116
100–299	242(27.91)	(<0.001)	438(50.52)	(<0.001)	551(63.55)	(<0.001)
300–499	274(28.96)		388(41.01)		531(56.13)	
500–999	1255(28.88)		2051(47.19)		2674(61.53)	
≥1000	332(21.42)		682(44.00)		874(56.39)	
Total	2103(27.28)		3559(46.17)		4630(60.06)	

Abbreviations: N/A, not applicable; ^a^, presented only as inpatient with psychiatric comorbidity; ^b^, presented only as inpatient with physical comorbidity; ^c^, presented only as inpatient with psychiatric or physical comorbidity.

**Table 2:** Prevalence of comorbid diseases by sub-diagnosis of mood disorders.

Mood Disorders (F30–F39)	Psychiatric Comorbidity ^a^		Physical Comorbidity ^b^		Total ^c^	
	*N*(%)	χ^2^ (*p*)	*N*(%)	χ^2^ (*p*)	*N*(%)	χ^2^ (*p*)
Manic episode (F30)	9(20.00)	141.173	21(46.67)	137.635	26(57.78)	221.953
Bipolar affective disorders (F31)	452(18.72)	(<0.001)	879(36.41)	(<0.001)	1154(47.80)	(<0.001)
Depressive episode (F32)	1321(31.12)		2151(50.67)		2788(65.68)	
Recurrent depressive disorders (F33)	249(30.11)		427(51.63)		544(65.78)	
Persistent mood disorders (F34)	49(41.88)		54(46.15)		80(68.38)	
Other mood disorders (F38)	3(42.86)		4(57.14)		4(57.14)	
Unspecified mood disorders (F39)	20(37.04)		23(42.59)		34(62.96)	
Total	2103(27.28)		3559(46.17)		4630(60.06)	

^a^, presented only as inpatient with psychiatric comorbidity; ^b^, presented only as inpatient with physical comorbidity; ^c^, presented only as inpatient with psychiatric or physical comorbidity.

**Table 3:** Analysis of association rules to comorbidity in patients with mood disorders.

Rules	*N*	Support	Confidence	Lift	IS Scale
Mood disorders (*n* = 7709)					
E10–E14→I10–I15	262	0.034	0.504	4.635	0.397
I10–I15→E10–E14	262	0.034	0.313	4.635	0.397
E70–E90→I10–I15	96	0.012	0.478	4.394	0.234
I10–I15→E70–E90	96	0.012	0.115	4.394	0.234
I10–I15→K20–K31	96	0.012	0.115	1.821	0.151
Manic episode (*n* = 45)					
R50–R69→H00–H06	1	0.022	1.000	45.000	1.000
H00–H06→R50–R69	1	0.022	1.000	45.000	1.000
D60–D64→I30–I52	1	0.022	1.000	45.000	1.000
I30–I52→D60–D64	1	0.022	1.000	45.000	1.000
D60–D64→R00–R09	1	0.022	1.000	45.000	1.000
Bipolar affective disorders (*n* = 2414)					
I10–I15→E10–E14	38	0.016	0.309	6.970	0.331
E10–E14→I10–I15	38	0.016	0.355	6.970	0.331
Depressive episode (*n* = 4245)					
G30–G32→F00–F09	54	0.013	0.844	19.361	0.496
F00–F09→G30–G32	54	0.013	0.292	19.361	0.496
E10–E14→I10–I15	182	0.043	0.555	4.206	0.425
I10–I15→E10–E14	182	0.043	0.325	4.206	0.425
I60–I69→I10–I15	46	0.011	0.575	4.359	0.217
Recurrent depressive disorders (*n* = 827)					
G30–G32→F00–F09	13	0.016	0.867	21.719	0.584
F00–F09→G30–G32	13	0.016	0.394	21.719	0.584
E70–E90→I10–I15	20	0.024	0.690	4.354	0.324
I10–I15→E70–E90	20	0.024	0.153	4.354	0.324
E70–E90→E10–E14	9	0.011	0.310	3.468	0.194
Persistent mood disorders (*n* = 117)					
E10–E14, I10–I15→G20–G26	2	0.017	0.667	39.000	0.816
E00–E07→N30–N39	2	0.017	0.667	26.000	0.667
N30–N39→E00–E07	2	0.017	0.667	26.000	0.667
G20–G26→E10–E14	2	0.017	1.000	19.500	0.577
E10–E14→G20–G26	2	0.017	0.333	19.500	0.577
Other mood disorders (*n* = 7)					
A15–A19→C15–C26	1	0.143	1.000	7.000	1.000
C15–C26→A15–A19	1	0.143	1.000	7.000	1.000
A15–A19→I10–I15	1	0.143	1.000	7.000	1.000
I10–I15→A15–A19	1	0.143	1.000	7.000	1.000
A15–A19→K70–K77	1	0.143	1.000	7.000	1.000
Unspecified mood disorders (*n* = 54)					
B00–B09→K00–K14	1	0.019	1.000	54.000	1.000
K00–K14→B00–B09	1	0.019	1.000	54.000	1.000
D10–D36→M40–M54	1	0.019	1.000	54.000	1.000
M40–M54→D10–D36	1	0.019	1.000	54.000	1.000
I30–I52→R00–R09	1	0.019	1.000	54.000	1.000

Abbreviations: IS, interest support; ICD-10 code: A15–A19, tuberculosis; B00-B09, viral infections characterized by skin and mucous membrane lesions; C15–C26, digestive organs; D10–D36, benign neoplasms; D60–D64, aplastic and other anemias; E00–E07, disorders of thyroid gland; E10–E14, diabetes mellitus; E70–E90, metabolic disorders; F00–F09, organic, including symptomatic, mental disorders; G20–G26, extrapyramidal and movement disorders; G30–G32, other degenerative diseases of the nervous system; H00–H06, disorders of eyelid, lacrimal system, and orbit; I10–I15, hypertensive diseases; I30–I52, other forms of heart disease; I60–I69, cerebrovascular diseases; K00–K14, diseases of oral cavity, salivary glands, and jaws; K20–K31, diseases of esophagus, stomach, and duodenum; K70–K77, diseases of liver; M40–M54, dorsopathies; N30–N39, other diseases of the urinary system; R00–R09, symptoms and signs involving the circulatory and respiratory systems; R50–R69, general symptoms and signs.

## Data Availability

Restrictions apply to the availability of these data. Data were obtained from KCDC and are available from https://www.cdc.go.kr/contents.es?mid=a20303010502 (accessed on 3 August 2020).
